# Supplementary material for: COZOID: contact zone identifier for visual analysis of protein-protein interactions
Source: BMC Bioinformatics. 2018 Apr 6;19:125. doi: 10.1186/s12859-018-2113-6 (PMC5889581; doi:10.1186/s12859-018-2113-6)
Supplement: Supplementary file 2 — Software build. Executable binary file of the software tool. (ZIP 118,784 kb) [file 12859_2018_2113_MOESM2_ESM.zip › cozoid/java/docs/beansbinding-1.2.1-doc.zip!/org/jdesktop/beansbinding/class-use/Binding.html]

Uses of Class org.jdesktop.beansbinding.Binding (Beans Binding)


---


|  |  |  |
| --- | --- | --- |
| **Overview** \| **Package** \| **Class** \| **Use** \| **Tree** \| **Deprecated** \| **Index** \| **Help** | |  |
| PREV   NEXT | **FRAMES**    **NO FRAMES**     **All Classes** |  |


---


## **Uses of Class org.jdesktop.beansbinding.Binding**

| Packages that use Binding | |
| --- | --- |
| **org.jdesktop.beansbinding** | Provides support for defining properties and creating bindings between sets of two properties. |
| **org.jdesktop.swingbinding** | Provides support for binding to complex Swing components, and documentation on the interesting Swing properties to bind to. |
| **org.jdesktop.swingbinding.impl** | Private implementation details for Swing binding. |

Uses of Binding in org.jdesktop.beansbinding

| Subclasses of Binding in org.jdesktop.beansbinding | |
| --- | --- |
| `class` | `AutoBinding<SS,SV,TS,TV>`             An implementation of `Binding` that automatically syncs the source and target by refreshing and saving according to one of three update strategies. |

| Methods in org.jdesktop.beansbinding that return Binding | |
| --- | --- |
| `Binding` | `BindingGroup.getBinding(java.lang.String name)`             Returns the `Binding` in this group with the given name, or `null` if this group doesn't contain a `Binding` with the given name. |

| Methods in org.jdesktop.beansbinding that return types with arguments of type Binding | |
| --- | --- |
| `java.util.List<Binding>` | `BindingGroup.getBindings()`             Returns a list of all `Bindings` in this group. |

| Methods in org.jdesktop.beansbinding with parameters of type Binding | |
| --- | --- |
| `void` | `BindingGroup.addBinding(Binding binding)`             Adds a `Binding` to this group. |
| `void` | `BindingListener.bindingBecameBound(Binding binding)`             Notification that a `Binding` has been bound. |
| `void` | `AbstractBindingListener.bindingBecameBound(Binding binding)`             Notification that a `Binding` has been bound. |
| `void` | `BindingListener.bindingBecameUnbound(Binding binding)`             Notification that a `Binding` has been unbound. |
| `void` | `AbstractBindingListener.bindingBecameUnbound(Binding binding)`             Notification that a `Binding` has been unbound. |
| `void` | `BindingGroup.removeBinding(Binding binding)`             Removes a `Binding` from this group. |
| `void` | `BindingListener.sourceChanged(Binding binding, PropertyStateEvent event)`             Notification that the source property of a `Binding` has fired a `PropertyStateEvent` for the `Binding's` source object. |
| `void` | `AbstractBindingListener.sourceChanged(Binding binding, PropertyStateEvent event)`             Notification that the source property of a `Binding` has fired a `PropertyStateEvent` for the `Binding's` source object. |
| `void` | `AbstractBindingListener.sourceEdited(Binding binding)`             **Deprecated.** *This method has been replaced by `AbstractBindingListener.sourceChanged(org.jdesktop.beansbinding.Binding, org.jdesktop.beansbinding.PropertyStateEvent)` and it will go away soon. It is being kept for a short period only, to assist in migration.* |
| `void` | `BindingListener.synced(Binding binding)`             Notification that the source and target of a `Binding` have been made in sync. |
| `void` | `AbstractBindingListener.synced(Binding binding)`             Notification that the source and target of a `Binding` have been made in sync. |
| `void` | `BindingListener.syncFailed(Binding binding, Binding.SyncFailure failure)`             Notification that the `Binding` attempted to sync the source and target, but the sync failed. |
| `void` | `AbstractBindingListener.syncFailed(Binding binding, Binding.SyncFailure failure)`             Notification that the `Binding` attempted to sync the source and target, but the sync failed. |
| `void` | `BindingListener.targetChanged(Binding binding, PropertyStateEvent event)`             Notification that the target property of a `Binding` has fired a `PropertyStateEvent` for the `Binding's` target object. |
| `void` | `AbstractBindingListener.targetChanged(Binding binding, PropertyStateEvent event)`             Notification that the target property of a `Binding` has fired a `PropertyStateEvent` for the `Binding's` target object. |
| `void` | `AbstractBindingListener.targetEdited(Binding binding)`             **Deprecated.** *This method has been replaced by `AbstractBindingListener.targetChanged(org.jdesktop.beansbinding.Binding, org.jdesktop.beansbinding.PropertyStateEvent)` and it will go away soon. It is being kept for a short period only, to assist in migration.* |

Uses of Binding in org.jdesktop.swingbinding

| Subclasses of Binding in org.jdesktop.swingbinding | |
| --- | --- |
| `class` | `JComboBoxBinding<E,SS,TS>`             Binds a `List` of objects to act as the items of a `JComboBox`. |
| `class` | `JListBinding<E,SS,TS>`             Binds a `List` of objects to act as the elements of a `JList`. |
| `class` | `JListBinding.DetailBinding`             `DetailBinding` represents a binding between a property of the elements in the `JListBinding's` source `List`, and the values shown in the `JList`. |
| `class` | `JTableBinding<E,SS,TS>`             Binds a `List` of objects to act as the rows of a `JTable`. |
| `class` | `JTableBinding.ColumnBinding`             `ColumnBinding` represents a binding between a property of the elements in the `JTableBinding's` source `List`, and a column in the table. |

Uses of Binding in org.jdesktop.swingbinding.impl

| Subclasses of Binding in org.jdesktop.swingbinding.impl | |
| --- | --- |
| `class` | `AbstractColumnBinding` |

---


|  |  |  |
| --- | --- | --- |
| **Overview** \| **Package** \| **Class** \| **Use** \| **Tree** \| **Deprecated** \| **Index** \| **Help** | |  |
| PREV   NEXT | **FRAMES**    **NO FRAMES**     **All Classes** |  |


---
